# The Role of Phase-Contrast MRI in Diagnosing Cerebrospinal Fluid Flow Abnormalities

**DOI:** 10.7759/cureus.57114

**Published:** 2024-03-28

**Authors:** Govindarajan BR, Praveen K Sharma, Yashaswinii Polaka, Pujitha S, Paarthipan Natarajan

**Affiliations:** 1 Department of Radio-Diagnosis, Saveetha Medical College and Hospital, Saveetha Institute of Medical and Technical Sciences, Saveetha University, Chennai, IND

**Keywords:** psv, edv, csf flow, stroke volume, phase contrast mri

## Abstract

Background

Cerebrospinal fluid (CSF) dynamics play a crucial role in maintaining the homeostasis of the central nervous system (CNS). Any disruption in CSF flow can lead to various congenital and acquired conditions, impacting neurological function and overall health. This study aims to analyze the significance of phase-contrast MRI in evaluating abnormalities in CSF flow and its diagnostic utility in various CSF-related disorders. Phase contrast MRI has emerged as a valuable tool for evaluating CSF dynamics non-invasively by examining CSF flow characteristics such as pulsatile flow patterns, hyperdynamic or hypodynamic flow, and disruptions in CSF circulation. Alterations in CSF pulsatility and stroke volume can indicate changes in intracranial compliance, vascular resistance, or CSF production and absorption rates. The findings of this study will advance our understanding of CSF physiology and its relevance in neurological pathologies, potentially leading to improved patient outcomes and management approaches.

Materials and methods

The study involved 36 patients and was conducted as an observational, prospective study over 18 months (October 2020 to March 2022) at the Department of Radiology, Saveetha Medical College and Hospital, Chennai. We utilized a 1.5 T Philips Multiva MRI scanner by Philips Healthcare in Amsterdam, Netherlands. The study included patients with suspected CSF flow abnormalities and abnormal MRI findings (normal pressure hydrocephalus (NPH), age-related brain atrophy, aqueduct stenosis (AS), Chiari malformation type 1, syringomyelia, or arachnoid cyst), alongside control exhibiting normal neurological symptoms and MRI results. Exclusions involved individuals with febrile seizures, neurological diseases, cerebrovascular accidents, anti-convulsive medication use, cardiac arrhythmia, or MRI contraindications. Post-processing involved analyzing stroke volume (SV), peak systolic velocity (PSV), end diastolic velocity (EDV), and mean flux. Statistical analysis was conducted using the Statistical Package for the Social Sciences (IBM SPSS Statistics for Windows, IBM Corp., Version 24.0, Armonk, NY), employing the χ2-test for categorical variables and nonparametric tests like Mann-Whitney U and Kruskal-Wallis H-tests for quantitative variables. A p-value < 0.05 was considered significant.

Results

The 36 patients, aged 1 to 80 years, were referred by the neurology department and categorized into four subgroups based on clinical history and conventional MRI findings: NPH, AS, age-related brain atrophy, and a normal control group. MRI CSF flowmetry evaluation focused on PSV, PDV, and SV. We found peak diastolic velocity (PDV), PSV, and average blood velocity (ABV) to be significantly higher in NPH compared to the control group (PSV, EDV, and SV: 9.96 +/- 1.73, 4.72 +/- 0.62, and 63 +/- 12.88 for NPH versus 4.8 +/- 0.39, 3.21 +/- 0.55, and 20.72 +/- 5.7 for control, respectively; p = 0.000). Conversely, patients with age-related brain atrophy and AS exhibited lower values (1.6 +/- 0.44, 1.13 +/- 0.09, and 6.33 +/- 2.08 for AS, and 2.07 +/- 0.09, 1.62 +/- 0.33, and 6.8 +/- 2.16 for age-related brain atrophy versus control; p = 0.002).

Conclusion

MRI CSF flowmetry emerges as a rapid, accurate, and non-invasive diagnostic tool for various neurological disorders associated with abnormal CSF flow. Additionally, this technique may aid in selecting appropriate treatment strategies.

## Introduction

Cerebrospinal fluid (CSF) surrounds the brain and spinal cord, providing support and protection against external stress [1,2]. Normal CSF pressure ranges from 5 to 15 mmHg in adults and 10 to 100 mmHg in children under six years old [2]. Phase-contrast MRI is used to detect and measure the pulsatile flow of CSF, particularly in areas like the aqueduct of Sylvius and foramen magnum, where CSF flow fluctuates during systole [1,3].

Two types of phase-contrast MRI techniques are used: axial and sagittal. Axial velocity encoding provides precise quantitative analysis, while sagittal in-plane velocity encoding is more beneficial for qualitative analysis. Adjustments to imaging planes are made to evaluate communication between arachnoid cysts and subarachnoid CSF spaces, particularly detecting pulsatile flow at the cyst's neck to indicate communication [1,4].

Qualitative analysis is most useful for assessing communication between arachnoid cysts and subarachnoid CSF spaces [1]. Adjusting the imaging plane according to the anticipated point of communication helps detect pulsatile flow at the cyst's neck, indicating communication. The lack of such a signal suggests no communication [1,5]. Phase-contrast MRI images can be presented separately or in closed-loop cine format.

For CSF flow assessment, phase images are manually drawn into circular regions of interest (ROIs) to incorporate all pixels indicating flow at the aqueduct. Velocity and volume flow rates can be quantified directly from velocity-time curves and flow-time curves [1,6]. Phase-contrast MRI can determine communication between arachnoid cysts and CSF, aiding treatment decisions. It can also assess CSF flow irregularity due to tonsillar herniation in Chiari 1 malformation [7].

Normal pressure hydrocephalus (NPH) presents with gait disturbance, urine incontinence, and dementia, with normal CSF pressure. Imaging shows hydrocephalus, and phase-contrast MRI helps diagnose NPH and differentiate it from brain atrophy [1,8].

Idiopathic intracranial hypertension (IIH) causes raised intracranial pressure without clear etiology or hydrocephalus [1,9]. Phase-contrast MRI reveals differences in CSF flow between IIH patients and controls, suggesting its utility in monitoring IIH [1,10].

In Chiari malformation type I (CM-I), cerebellar tonsils are displaced, often with syringomyelia. Phase-contrast MRI assesses CSF flow disturbance severity, correlating with clinical symptoms [11].

Brain atrophy presents with ventricular dilation and increased white matter signal intensity on MRI, proportional to subarachnoid space enlargement [1].

This study aims to analyze the diagnostic significance of phase-contrast MRI in various CSF flow abnormalities and its role in understanding CSF-related diseases, potentially improving diagnostic and therapeutic strategies for patients with CSF-related disorders.

## Materials and methods

The study was conducted as a cohort study, prospectively at the Department of Radio-Diagnosis over a duration of 18 months, involving 36 patients, consisting of 18 cases with neurological symptoms and 18 controls referred from the Department of Neurology. Cases meeting specific inclusion criteria, showing suspected CSF flow abnormalities based on clinical manifestations and conventional MRI findings such as NPH, age-related brain atrophy, aqueductal stenosis (AS), CM-1, syringomyelia, and arachnoid cysts, were enrolled. Patients with neurological symptoms but normal conventional findings and normal CSF flow were considered controls, while those with exclusions such as complex febrile seizures, neurological diseases, cerebrovascular accidents, anti-convulsive medications, cardiac patients with arrhythmia, routine MRI contraindications, and those unwilling to participate were excluded.

Data collection utilized a stratified sampling method, with comprehensive evaluations conducted through history-taking and phase-contrast MRI protocols, encompassing both qualitative and quantitative assessments of CSF flow dynamics. Various examination protocols were employed to assess CSF flow properties and connectivity with subarachnoid spaces, utilizing specific imaging parameters and post-processing calculations. Patient data confidentiality was ensured, and any unanticipated risks during the research were promptly disclosed to participants and the ethics committee.

The study aimed to provide insight into the diagnostic utility of phase-contrast MRI in CSF-related diseases and to investigate CSF flow dynamics involvement in neurological conditions, facilitating more accurate diagnosis and management strategies.

The phase-contrast MRI protocol utilized a 1.5 T Philips Multiva MRI system (Philips Healthcare, Amsterdam, Netherlands) with a head coil, incorporating routine conventional MRI sequences and CSF flow studies such as CSF DRIVE (Dynamic Response Imaging of CSF Velocity), CSF QF (Quantitative Flow), and CSF PCA (Phase Contrast Angiography). Imaging parameters for T2 included TR 5000, TE 105, NEX 2, FA1/100, FOV 240, and matrix 224 x 384. Patients were positioned supine with a head-first orientation. Thin cuts in the mid-sagittal steady-state free precession sequence allowed for the accurate evaluation of the CSF flow void sign along the aqueduct of Sylvius. MR-compatible electrodes ensured cardiac gating, and sagittal T2-weighted images or an SSPS sequence provided localization on the cerebral aqueduct for CSF flow assessment. Two-dimensional cine phase-contrast MRI (2D cine PC-MRI) with cardiac gating employed specific imaging parameters including TR=25, TE=4.3, flip angle = 100°, and VENC ranging from 5-20 cm/s.

Examination protocols varied based on pathology, with analysis predominantly focused on the aqueduct of Sylvius. CSF flow quantification involved ROI measurements and post-processing calculations, including peak systolic velocity (PSV), end diastolic velocity (EDV), stroke volume (SV), average velocity, and flow rate. This comprehensive approach facilitated accurate assessment of CSF flow dynamics and pathology-specific characterization.

Post-processing calculations involved determining various parameters to characterize CSF flow dynamics. PSV (cm/sec) was identified as the highest CSF velocity measured during systole, while EDV (cm/sec) represented the highest CSF velocity measured during diastole. SV (μl) was calculated as the mean CSF volume flowing over the aqueduct during systole, obtained by multiplying mean flow by CSF duration during systole. Additionally, average velocity (cm/sec) for both systole and diastole was computed. Finally, flow rate (ml/min) was derived by multiplying the ROI area (cm^2) by mean velocity, providing a comprehensive understanding of CSF flow characteristics in the studied population.

## Results

In this study, conducted at the Department of Radio-Diagnosis, among the 36 patients, males were more than females representing 20 (55.6%) and 16 (44.4%) respectively (Table 1).

**Table 1:** Gender distribution of the study participants

GENDER	FREQUENCY (n=36)	PERCENTAGE (%)
Male	20	55.6
Female	16	44.4

There were patients ranging in age from 1 year to 80 years, 1-20 years (25%), 21-40 years (22.2%), 41-60 years (25%) and 61-80 years (27.8%) (Table 2).

**Table 2:** Age distribution of the study participants

AGE	FREQUENCY (n=36)	PERCENTAGE (%)
1-20 years	9	25
21-40 years	8	22.2
41-60 years	9	25
61-80 years	10	27.8

**Figure 1:**
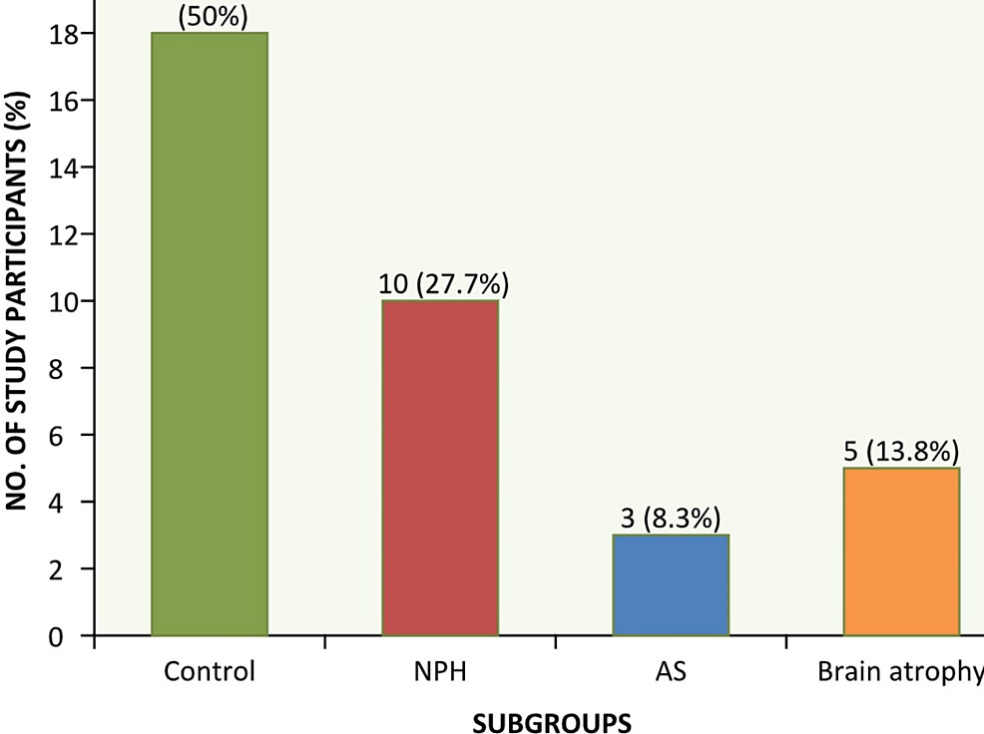
Frequency of study subgroups NPH: Normal pressure hydrocephalus, AS: Aqueduct stenosis

The study participants were sub-grouped into 18 control (50%) and 18 cases (50%). Among them 10 NPH (27.7%), three aqueductal stenosis (8.3%) and five patients with age-related brain atrophy (13.8%) (Figure 1).

The aqueductal CSF flow was assessed using cine phase-contrast MRI in four distinct groups: a control group, a group with NPH, a group with AS, and a group with age-related brain atrophy. The control group comprised 18 participants, consisting of 11 males and seven females, with a mean age of 28.06 ± 14.35 years. In contrast, the NPH group included 10 participants, with three males and seven females, exhibiting symptomatic ventriculomegaly, and had a mean age of 63.4 ± 7.07 years. Additionally, three patients diagnosed with aqueductal stenosis, including two males and one female, were assessed, with a mean age of 2.33 ± 0.57 years. Furthermore, five patients with age-related brain atrophy, comprising four males and one female, were evaluated, with a mean age of 66.20 ± 7.19 years.

A Kruskal-Wallis test was conducted to compare the aqueductal CSF flow among the different groups, yielding a test statistic of 26.74 and a p-value of <0.001, indicating a statistically significant difference in CSF flow among the groups. The association between cases and sex distribution was evaluated, revealing that female participants accounted for 43.8% of NPH cases and 6.3% of cases with AS and age-related atrophy. Although these percentages were higher compared to male participants, statistical analysis indicated that the differences were not statistically significant (p > 0.05) (Table 3). A chi-square test was performed to assess the association, yielding a test statistic of 4.23 and a p-value of 0.23, further confirming the lack of statistically significant association between cases and sex distribution (Table 3).

**Table 3:** Age and gender distribution among control and study subgroups SD: Standard deviation, NPH: Normal pressure hydrocephalus, AS: Aqueduct stenosis

AGE AND GENDER DISTRIBUTION	CONTROL	NPH (n=10)	AS (n=3)	AGE-RELATED BRAIN ATROPHY (n=5)	TOTAL (n=36)
Age - Mean ± SD	29.06 ± 14.3	63.40 ± 7.0	2.33 ± 0.5	66.20 ± 7.1	41.53 ± 2
Male gender	11 (55%)	3 (15%)	2 (10%)	4 (20%)	20 (55.6%)
Female gender	7 (43.8%)	7 (43.8%)	1 (63%)	1 (6.3%)	16 (44.4%)

The MRI CSF flowmetry evaluation focused on PSV, peak diastolic velocity (PDV), and SV in both cases and controls. In the NPH group, PSV was significantly higher compared to the control group (9.96 ± 1.73 vs. 4.80 ± 0.39, p < 0.05). Conversely, patients with AS exhibited lower PSV values compared to the control group (1.6 ± 0.44 vs. 4.80 ± 0.39, p < 0.05), as did patients with age-related brain atrophy (2.07 ± 0.09 vs. 4.80 ± 0.39, p < 0.05).

Additionally, comparisons between the control group and each of the NPH, AS, and age-related brain atrophy groups regarding PSV were conducted using the Mann-Whitney U-test. The results showed significant differences between the control and NPH groups (U-test result: 0.000, p-value: 0.000), as well as between the control and AS groups (U-test result: 54.000, p-value: 0.002). Similarly, a significant difference was observed between the control and age-related brain atrophy groups (U-test result: 90.000, p-value: 0.000) (Table 4).

**Table 4:** Comparison between control and study subgroups regarding PSV PSV: Peak systolic volume, SD: Standard deviation, NPH: Normal pressure hydrocephalus, AS: Aqueduct stenosis

PSV	CONTROL (n=18)	NPH (n=10)	AS (n=3)	AGE-RELATED BRAIN ATROPHY (n=5)
Mean ± SD	4.80 ± 0.39	9.96 ± 1.73	1.6 ± 0.44	2.07 ± 0.09
Median	4.86	10.13	1.49	2.1
Mode	4.11	7.50	1.23	2.10
Range	1.26 (4.11-5.37)	4.90 (7.5-12.4)	0.87 (1.23-2.10)	0.26 (1.94-2.20)

In the NPH group, end diastolic volume (EDV) was significantly higher compared to controls (4.72 ± 0.62 vs. 3.21 ± 0.55, p < 0.05). Conversely, patients with AS exhibited lower EDV values compared to controls (1.13 ± 0.09 vs. 3.21 ± 0.55, p < 0.05), as did patients with age-related brain atrophy (1.62 ± 0.33 vs. 3.21 ± 0.55, p < 0.05).

Furthermore, comparisons between the control group and each of the NPH, AS, and age-related brain atrophy groups regarding EDV were conducted using the Mann-Whitney U-test. The results revealed significant differences between the control and NPH groups (U-test result: 6.000, p-value: 0.000), as well as between the control and AS groups (U-test result: 54.000, p-value: 0.002). Similarly, a significant difference was observed between the control and age-related brain atrophy groups (U-test result: 90.000, p-value: 0.000) (Table 5).

**Table 5:** Comparison between control and study subgroups regarding EDV EDV: End diastolic volume, SD: Standard deviation, NPH: Normal pressure hydrocephalus, AS: Aqueduct stenosis

EDV (cm/sec)	CONTROL (n=18)	NPH (n=10)	AS (n=3)	AGE-RELATED BRAIN ATROPHY (n=5)
Mean ± SD	3.21 ± 0.55	4.72 ± 0.62	1.13 ± 0.09	1.62 ± 0.33
Median	3.31	4.93	1.12	1.57
Mode	3.01	5.39	1.05	1.23
Range	1.67 (2.23-3.90)	1.80 (3.59-5.39)	0.18 (1.05-1.23)	0.87 (1.23-2.1)

In the NPH group, SV was significantly higher compared to controls (63 ± 12.88 vs. 20.72 ± 5.7, p < 0.05). Conversely, patients with AS exhibited lower SV values compared to controls (6.33 ± 2.08 vs. 20.72 ± 5.7, p < 0.05), as did patients with age-related brain atrophy (6.8 ± 2.16 vs. 20.72 ± 5.7, p < 0.05).

Furthermore, comparisons between the control group and each of the NPH, AS, and age-related brain atrophy groups regarding SV were conducted using the Mann-Whitney U-test. The results revealed significant differences between the control and NPH groups (U-test result: 0.000, p-value: 0.000), as well as between the control and AS groups (U-test result: 54.000, p-value: 0.002). Similarly, a significant difference was observed between the control and age-related brain atrophy groups (U-test result: 90.000, p-value: 0.000) (Table 6).

**Table 6:** Comparison between control and study sub-groups regarding SV SV: Stroke volume, SD: Standard deviation, NPH: Normal pressure hydrocephalus, AS: Aqueduct stenosis

SV (cm/sec)	CONTROL (n=18)	NPH (n=10)	AS (n=3)	AGE-RELATED BRAIN ATROPHY (n=5)
Mean ± SD	20.72 ± 5.7	63 ± 12.88	6.33 ± 2.08	6.8 ± 2.16
Median	20.50	60	7.00	7.00
Mode	22	42	4	7
Range	20 (11-31)	39 (42-81)	4 (4-8)	6 (4-10)

## Discussion

In a comparison between our study and the research conducted by Battal et al. [12], we found both similarities and differences across various groups. While Battal et al. had a control group of 10 participants, our study included 18 participants in the control group, showing a similar age distribution (22.75 +/- 19.53 in Battal et al. vs. 29.06 +/- 14.35 in our study). Both studies exhibited similar mean PSV, EDV, and SV values in the control group.

Regarding the NPH groups, Battal et al. recorded slightly lower PSV, EDV, and SV values compared to our findings. However, our study revealed a clear increase in systolic flow and SV, indicating hyperdynamic circulation similar to Battal et al. The SV in our NPH group was notably larger than that in the control group, showing statistical significance (p-value < 0.05). This aligns with previous research by Kahlon et al., which noted significant differences between NPH and control groups [13]. Moreover, our observation of clinical improvement post-shunt surgery in patients with hyperdynamic CSF flow corroborates Bradley's findings, indicating a higher aqueductal SV in these patients compared to healthy older individuals [14].

Furthermore, phase-contrast MRI examinations conducted on NPH patients revealed significantly greater values in all parameters compared to the control group, consistent with findings by Ahmed et al. [15] and Giner et al. [16]. The utility of this technique in hydrocephalus classification, particularly in distinguishing communicating from non-communicating hydrocephalus, was underscored.

Given the overlapping clinical presentations of NPH and brain atrophy, our study addressed distinguishing between these conditions. We found significantly lower SVs in patients with atrophy compared to healthy controls, indicating hypodynamic circulation, consistent with prior research by Battal et al. [12]. This was statistically significant (p-value < 0.05). Similarly, MRI with phase contrast in age-related brain atrophy patients revealed substantial declines in all parameters compared to the control group, further supporting the presence of hypodynamic CSF flow in atrophy.

In the AS group, PSV and EDV values aligned closely with Battal et al.'s findings [12]. Our evaluation of AS patients demonstrated lower velocities compared to the control group, consistent with Kim et al.'s study on 16 individuals, which reported maximal systolic velocity of the aqueductal CSF flow to be less than 1 cm/sec [17].

In summary, our research parallels Battal et al.'s findings in control and patient groups, highlighting significant differences in CSF flow characteristics across various conditions, particularly NPH and brain atrophy, and affirming the diagnostic utility of phase-contrast MRI in hydrocephalus classification and etiological differentiation [12].

Benefits of this study include differentiation of congenital and acquired conditions that affect CSF dynamics and assess the effectiveness of phase contrast MRI in detecting CSF flow anomalies. Additionally, it seeks to correlate CSF pulsatility and SV with various CSF-related disorders, potentially identifying biomarkers for early diagnosis and treatment monitoring. This study has the potential to enhance diagnostic accuracy, refine imaging protocols, and improve patient care in neurology.

Limitations of our study include conducting it exclusively in a single tertiary care hospital, which may limit the generalizability of our findings to broader populations. The limited number of study individuals was primarily due to the affordability of the procedures involved, which constrained our ability to recruit a larger and more diverse sample.

## Conclusions

Cine phase-contrast MRI is a valuable imaging method for assessing CSF dynamics, which can influence various disease processes. This study holds paramount importance in advancing our understanding and management of neurological disorders by investigating the role of CSF dynamics using phase-contrast MRI. By detecting abnormalities in CSF flow, the study aims to enable early diagnosis of conditions such as hydrocephalus, CM, and arachnoid cysts, facilitating timely intervention and preventing disease progression. The importance of this study is that, in patients with NPH, a positive response to shunting has been associated with CSF pulsatility and SV across the aqueduct. It can differentiate between different posterior fossa cystic abnormalities and assess the effectiveness of surgical procedures. Moreover, the presence of pulsatile CSF flow within cystic cord lesions can help distinguish between a syrinx and myelomalacia. MRI CSF flowmetry offers a quick and painless method to diagnose and monitor various neurological conditions that may cause abnormal CSF flow. Additionally, it can differentiate between NPH and brain atrophy. Ultimately, the goal is to enhance patient care by optimizing diagnosis and treatment based on CSF dynamics, thereby improving the quality of life for individuals affected by these neurological disorders.
